# Supportive care interventions for managing gastrointestinal symptoms following treatment for colorectal cancer: a systematic review

**DOI:** 10.1007/s11764-023-01403-3

**Published:** 2023-06-06

**Authors:** Angela Ju, Lisette Wiltink, Jared Walker, Kate White, Claudia Rutherford

**Affiliations:** 1 School of Psychology, Sydney Quality of Life Office, The University of Sydney, Sydney, 2006 Australia; 2 Department of Radiation Oncology, Leiden University Medical Center, Leiden, The Netherlands; 3 Faculty of Medicine, University of Queensland, Brisbane, Australia; 4 Faculty of Medicine and Health, Susan Wakil School of Nursing and Midwifery, Cancer Care Research Unit (CCRU), The University of Sydney, Sydney, Australia; 5 The Daffodil Centre, The University of Sydney, a joint venture with Cancer Council NSW, Sydney, Australia

**Keywords:** Bowel cancer, Systematic review, Interventions, Symptoms, Functioning

## Abstract

**Introduction:**

Colorectal cancer (CRC) is prevalent in the developed world, with unhealthy lifestyles and diet contributing to rising incidence. Advances in effective screening, diagnosis, and treatments have led to improved survival rates, but CRC survivors suffer poorer long-term gastrointestinal consequences than the general population. However, the current state of clinical practice around provision of health services and treatment options remains unclear.

**Purpose:**

We aimed to identify what supportive care interventions are available to manage gastrointestinal (GI) symptoms for CRC survivors.

**Methods:**

We searched Cochrane Central Register of Controlled Trials, Embase, MEDLINE, PsycINFO, and CINAHL from 2000 to April 2022 for resources, services, programs, or interventions to address GI symptoms and functional outcomes in CRC. We extracted information about characteristics of supportive care interventions, the study design, and sample characteristics from included studies, and performed a narrative synthesis

**Results:**

Of 3807 papers retrieved, seven met the eligibility criteria. Types of interventions for managing or improving GI symptoms included two rehabilitation, one exercise, one educational, one dietary, and one pharmacological. Pelvic floor muscle exercise may help to resolve GI symptoms more quickly in the post-operative recovery phase. Survivors may also benefit from rehabilitation programs through improved self-management strategies, especially administered soon after completing primary treatment.

**Conclusions/Implications for cancer survivors:**

Despite a high prevalence and burden of GI symptoms post-treatment, there is limited evidence for supportive care interventions to help manage or alleviate these symptoms. More, large-scale randomized controlled trials are needed to identify effective interventions for managing GI symptoms that occur post-treatment.

**Supplementary Information:**

The online version contains supplementary material available at 10.1007/s11764-023-01403-3.

## Background

Colorectal cancer (CRC), including bowel, colon, and rectal cancer, is the fourth most common cancer comprising 11% of all cancer diagnoses [1]. Globally, incidence of CRC is expected to increase to over 2.2 million by the year 2030, due to sedentary lifestyles, greater intake of processed foods and alcohol, and obesity [2]. Although CRC has the third highest mortality rate, advances in screening, early diagnosis, and treatments for CRC have led to increased survival rates. Overall 5-year survival rate is 64–69% but can be as high as 90% if diagnosed at a localized stage [3].

Despite longer life years gained, CRC survivors suffer from long-term persistent or late effects of treatment, with gastrointestinal (GI) symptoms among the most frequent [4]. Such symptoms can be assessed and monitored through patient-reported outcomes (PROs), which provide a way to quantitatively capture a patient’s perception of their own health [5]. PROs for GI symptoms can include excessive flatulence, abdominal pain, bloating, bowel function (e.g. constipation and diarrhoea), faecal incontinence, and nausea [6]. Two to 3 years after primary treatment, only 12% of survivors were satisfied with their defecation function and up to 63% of survivors experienced faecal incontinence [7]. Bowel dysfunction has been reported to be an ongoing problem even 15 years after diagnosis [8].

Although there is an urgent need for strategies to treat and manage ongoing physical consequences of treatment, CRC survivors seem ill-prepared for the long-term consequences, and reports from survivors suggest these effects are underestimated by clinicians [9]. Patients report the need for timely, relevant, and tailored information about, and interventions to, manage GI symptoms [10]. The available evidence suggests that survivors find ways to self-manage their GI symptoms and functioning impairments rather than seek professional help [10]. The reason for this may be multifactorial, owing to the paucity of interventions available and knowledge or access to existing services and supports.

Currently, little is known about what health services and interventions are available to manage physical symptoms and functions following treatment for CRC. Evidence is needed about health services and interventions that effectively manage GI symptoms to replace the long and often painful process of trial and error and to reduce unnecessary suffering.

This study aimed to identify and describe interventions available for managing GI symptoms in individuals who have received treatment for CRC (referred to as CRC survivors hereon in). Specifically, this study will determine the following: (1) what interventions are available for CRC survivors to manage GI symptoms post-treatment and their effectiveness, (2) provide characteristics of the available interventions, and (3) raise awareness of any gaps in intervention availability.

## Methods

We conducted a systematic review of clinical trials of interventions for managing or improving GI symptoms following treatment for CRC. The Preferred Reporting Items for Systematic Reviews and Meta-Analyses (PRISMA) checklist was used to guide the standards of reporting for this review(REF).

### Electronic searches

We searched Cochrane Central Register of Controlled Trials, Embase, MEDLINE, and PsycINFO from 2000 to April 2022. The date restriction was selected to ensure relevance of findings as surgical treatments, which is the main treatment for CRC, has evolved in the past 10–15 years. Our search strategy comprised a comprehensive set of terms for “patient-reported outcome,” “bowel cancer,” “intervention,” and “service” (sample search included as Supplementary File 1). We supplemented electronic searches by searches of the reference lists of the included studies and other related review papers.

### Study selection and eligibility criteria

References were included if they:

- Evaluated any intervention designed to address patient-reported GI symptoms in CRC survivors arising in the post-treatment survivorship phase and;
- Greater than or equal to 75% of the study sample consisted of CRC survivors if pooled sample in analysis or reported results separately for CRC survivors

Studies were excluded if:

- Interventions were designed for paediatric population or adult populations other than CRC survivors
- Studies described or evaluated preventative interventions delivered prior to or concurrently with primary treatment to prevent a symptom or impairment
- Studies describing or evaluating primary treatment for CRC (e.g. surgery, chemotherapy)
- Non-primary research (e.g. abstracts, protocols, reviews)

Retrieved titles and abstracts were independently screened for eligibility by two reviewers (NF and AJ); 25% of the retrieved citations were selected at random and were cross-checked by a third reviewer (LW). Where abstracts met eligibility or relevance was ambiguous, papers were obtained and reviewed in full. Full texts were independently reviewed by two reviewers (AJ and JW). Disagreements were resolved through team discussion.

### Data extraction and synthesis

A data extraction form was developed, including study aim, sample demographics (e.g. age, gender), tumour characteristics (e.g. stage and type), characteristics of intervention (e.g. frequency of administration, duration), follow up period, and results of patient reported GI symptoms. Characteristics of interventions are described below. Narrative synthesis was used to summarize intervention characteristics and their effectiveness for managing GI symptoms.

## Results

### Summary of included studies

**Fig. 1:**
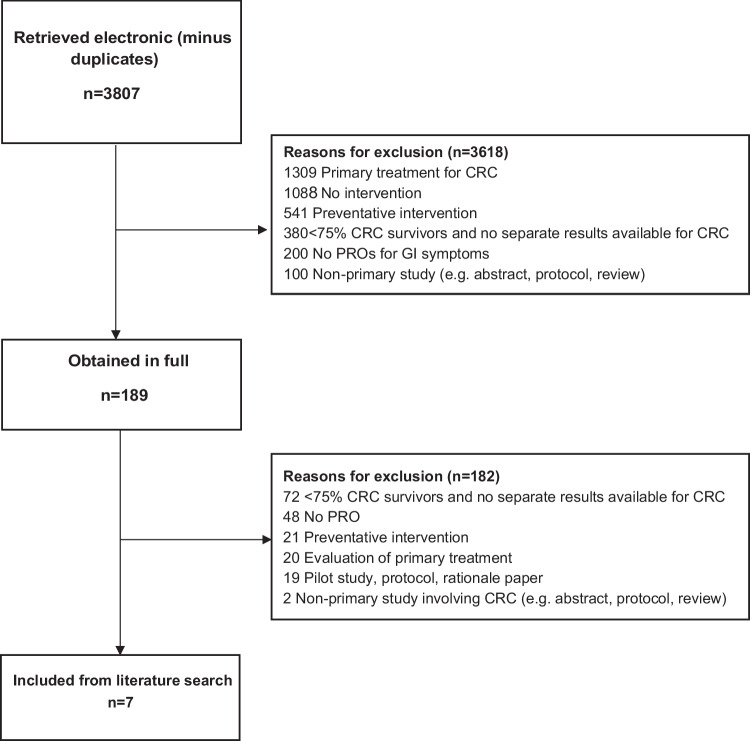
Flowchart of included paper systematic review GI interventions

The search yielded 3807 papers, of which six met the eligibility criteria, four RCTs, and two pilot studies designed to inform larger RCTs (Fig. 1). Across studies, 319 CRC survivors who had undergone surgery as their primary treatment for CRC were included. Intervention types included five exercise, two rehabilitation, one educational, one dietary, and one pharmacological. Characteristics of included studies and study results are summarized in Tables 1 and 2, respectively.

**Table 1:** Characteristics of included studies

Author (year), country	Intervention sample size, sex ratio M:F, age mean (SD, range), cancer type and stage, n ostomy permanent	Comparison sample size, sex ratio M:F, age mean (SD, range), cancer type and stage, n ostomy permanent	Primary treatment for CRC	Intervention type and comparison group, description and duration	Primary PRO	PRO assessment timepoints
Hung et al. (2016) [17], Taiwan	Sample size: 26 18:8 Mean age: 66.8 (12.5, 27–79) Cancer type: CRC (n26) Cancer stage: 1 (n9), 2 (n9), 3 (n7), 4 (n1) N permanent ostomy: n0	Sample size: 26 24:2 Mean age: 66.8 (12.5, 27-79) Cancer type: CRC (n26) Cancer stage:1 (n6), 2 (n12), 3 (n5), 4 (n3) N permanent ostomy: n0	Colostomy closure and coloanal anastomosis surgery	Pelvic floor muscle training (PFMT) exercises vs. standard care Description: 20 PFM contractions and relaxations 4 times/day, self-administered Duration: 9 months	Bowel Function	T0: Baseline T1: 1 month T2: 2 months T3: 3 months T4: 6 months T5: 9 months
Park et al. (2007) [22], South Korea	Sample size: 17 12:5 Mean age: 61.0 (9.7) Cancer type: RC (n17) Cancer stage: 1 (n9), 2 (n5), 3 (n2), 4 (n1) N permanent ostomy: nr~	Sample size: 12 6:6 Mean age: 59.5 (9.0) Cancer type: RC (n12) Cancer stage: 1 (n5), 2 (n2), 3 (n5), 4 (n0) N permanent ostomy: nr~	Low anterior resection	30% Phenylephrine gel vs. control Description: Gel applied twice daily for 4 weeks, administration NR Duration: 4 weeks	Bowel function	T0: Baseline T1: 4 weeks
Stephens et al. (2012) [20], Australia	Sample size: 18 14:4 Mean age: 1.95 (13.91) Cancer type: RC (n18) Cancer stage: nr N permanent ostomy: n0	Sample size: 20 10:10 Mean age: 63.34 (9.06) Cancer type: RC (n20) Cancer stage: nr N permanent ostomy: n0	Anterior resection or Hartmann’s procedure	2.5g Lactic acid bacteria (VSL#3 sachet) vs. control Description: take one packet twice daily, for 4 weeks, self-administered Duration: 4 weeks	Bowel function	T0: Baseline (pre-op) T1: 1 week T2: 2 weeks T3: 3 weeks T4: 4 weeks
Li et al. (2022) [18], China	Sample size: 47 24:23 Mean age: 60.5 (9.2) Cancer type: RC Cancer stage: 1 (n18), 2 (n17), 3 (n11), 4 (n1) N permanent ostomy: 26	Sample size: 48 28:20 Mean age: 61.3 (10.2) Cancer type: RC Cancer stage: 1 (n14), 2 (n14), 3 (n17), 4 (n4) N permanent ostomy: 29	Sphincter preserving surgery	Self-management program vs standard care. Description: 30–60 min face-to-face self-management program post-operatively and follow-up telephone appointments every 1–2 weeks. Duration: 6 months	Bowel function	T0: Baseline T1: 3 months T2: 6 months
Lin et al. (2019) [19], Australia	Sample size: 10 7:3 Mean age: 70.0 (6.2) Cancer type: CRC (n10) Cancer stage: 1 (n3), 2 (n2), 3 (n5), 4 (n0) N permanent ostomy: 0	Sample size: 10 M:F NR Mean age: 69.0 (12.8) Cancer type: CRC (n10) Cancer stage: 1 (n3), 2 (n3), 3 (n3), 4 (n), missing (1) N permanent ostomy: 0	Variable (3 right and 2 left hemicolectomy, 2 low anterior resection, 1 high anterior resection, 2 ultra-low anterior resection)	Multi-disciplinary education and exercise program vs control group. Description: comprises dietetics, physiotherapy, exercise physiology and psychology vs control group. Duration: 8-weeks	Pelvic floor symptoms, incontinence	T0: Baseline T1: 18 weeks T2: 6 months
Sun et al. (2022) [25], USA	Sample size: 10 Cancer type: CRC Cancer stage: 1, 2, and 3	N/A	Variable (any surgery, chemotherapy, radiation therapy) with permanent ostomy or anastomosis	Telephone-based diet and behaviour change intervention vs control group. Description: involves dietary recall, food and symptom diaries, motivational interviewing, and health coaching. Duration: 10 sessions over 9–10 months	Bowel function	T0: Baseline (6 months post treatment) T1: 4 months T2: 6 months

**Table 2:** Results of included studies

Author (year), country	Primary patient-reported outcome measure for gastrointestinal symptom	Baseline before intervention			Last follow-up time point after intervention			
		Intervention group M (SD)	Control group *M* (SD)	P value	Intervention group *M* (SD)	Control group *M* (SD)	*P* value	Group effect *P* value
Hung et al. (2016) [17], Taiwan	Fecal incontinence quality of life (FIQL) score	Median = 13.62 (2.50)	Median = 12.50 (2.93)	0.14	Median = 14.44 (1.85)	Median = 14.67 (2.06)	0.03	0.14
Park et al. (2007) [22], South Korea	Fecal incontinence severity index (FISI) score	32.5 (14.50)	32.3 (14.70)	0.94	32.1 (11.20)	32.4 (14.40)	0.63	
Stephens et al. (2012) [20], Australia	Gastrointestinal quality of life index (GQLI) score	107.67 (17.76)	109.55 (17.98)	0.75	111.61 (16.02)	106.40 (17.52)	0.35	
Li et al. (2022) [18], China	Low anterior resection syndrome (LARS) score	31.60 (SE = 9.10)	28.20 (SE = 10.00)	-	22.90 (SE = 10.60)	22.80 (SE = 11.80)	-	0.580; time effect *p* value <.001
Lin et al. (2019) [19], Australia	APFQ bowel	2.27 (1.49)	1.77 (0.89)	-	1.94 (1.29)	1.47 (0.42)	-	0.33
	ICIQ-B bowel control	7.40 (6.96)	4.00 (5.03)	-	4.70 (4.74)	3.14 (3.72)	-	0.10
	ICIQ-B bowel pattern	8.20 (4.77)	7.20 (3.46)	-	6.50 (2.99)	6.43 (3.15)	-	0.47
Sun et al. (2022) [25], USA	Lower anterior resection syndrome (LARS) score	Not reported	Not reported		Not reported	Not reported		

*M (SD)* mean (standard deviation), unless otherwise stated; *SE* standard error

### Assessment of PROs

Self-reported GI symptoms were assessed using six different patient-reported outcome measures (PROMs) (Table 3).

**Table 3:** PROMs used in included studies

Study	PROM*	Domains
Hung (2016)	Fecal incontinence quality of life scale [11]	Impact of fecal incontinence on lifestyle, coping/behaviour, depression/self-perception and embarrassment
Li (2022)	Low anterior resection syndrome score [12]	Control of flatus, accidental leakage of liquid stool, urgency, frequency of bowel movements
Lin (2019)	Australian pelvic floor questionnaire [13]	Bladder function, bowel function, prolapse symptoms, sexual function
	International Consultation on Incontinence Questionnaire-Bowel module [14]	Bowel pattern, bowel control, and quality of life,
Park (2007)	Fecal incontinence severity index [15]	Frequency and type of incontinence (e.g. gas, liquid, solid stool, mucous)
Stephens (2012)	Gastrointestinal quality of life index [16]	GI symptoms (e.g. abdominal pain, bloating, excessive gas, frequency of bowel movements, belching, diet), emotion, physical function, social function, medical treatment
Sun (2022)	Low anterior resection syndrome score [12]	Control of flatus, accidental leakage of liquid stool, urgency, frequency of bowel movements

*Used to assess primary outcome

### Types of interventions

#### Exercise programs

One study evaluated a pelvic floor muscle exercise (PFME) program compared to standard, post-operative care following colostomy closure and coloanal anastomosis surgery [17]. Standard care consisted of a pamphlet outlining post-surgical care such as wound management and diet. Bowel symptoms gradually improved over 12 months for both routine care and PFME groups. However, survivors participating in the PFME program reported significantly quicker improvement in faecal incontinence than those receiving standard care. The PFME group also demonstrated a significantly quicker reduction in GI quality of life index scores, which encompasses symptoms such as abdominal pain, bloating, diet, and bowel movements [16].

#### Comprehensive rehabilitation

Two studies evaluated a comprehensive rehabilitation program following stoma reversal [18, 19]. Such programs comprised (1) an educational component (e.g. diet, perianal skin care, defecation reflex, posture), (2) exercise (e.g. increasing physical activity, PFME), and (3) psychosocial (e.g. coping mechanisms, mood management). Standard care included (1) discharge education by a clinical nurse about diet, medication, dressing changes, and outpatient follow-up booking and (2) routine phone call within a month after discharge to check wound healing and diet [18]. Both studies reported a significant improvement in patient-reported bowel symptoms at the time completion of the program, which were maintained at 6-month follow-up.

#### Diet

One study evaluated the effect of adding the probiotic VSL #3 to regular diet of CRC survivors who had undergone a reversal of their loop ileostomy [20]. VSL #3 has been shown to improve bowel symptoms in other conditions such as ulcerative colitis and Crohn’s disease through altering micro-ecology of the colon. However, probiotic VSL #3 did not show any significant benefit in post-operative bowel symptoms and other GI symptoms such as abdominal pain and frequency of bowel movements, as measured by GI quality of life index [16] throughout and at the end of the 4-week intervention period.

Another study investigated the acceptability of a novel, telehealth dietary education program for CRC survivors at least 6 months after their primary surgical treatment [21]. The program consisted of 10 sessions, comprising of (1) educational content — diet recommendations for cancer survivorship, problem-solving for symptoms, and goal setting and (2) participant activities — keeping a food and symptom diary and eliminating or substituting food groups. The program was perceived as acceptable and feasible for CRC survivors and demonstrated potential to improve urgency and incontinence at 4 and 6 months following the completion of the program compared to baseline.

#### Pharmacology

One study examined the effect of phenylephrine gel on ongoing anal incontinence following low anterior resection [22]. Phenylephrine is a selective alpha-1 adrenergic agonist, which has been shown to cause internal and sphincteric contractions in in vitro studies. However, at the end of the 4-week treatment, no significant difference was observed in severity of faecal incontinence between placebo and phenylephrine groups.

### Quality assessment

Quality assessment for RCTs were conducted using the Consolidated Standards of Reporting Trials (CONSORT) checklist(REF). Studies were given a score of 1 for each item that was reported, and 0 if it was not reported. The total score was converted to a percentage to aid comparison. RCT quality scores (*n* = 5) ranged from 61 to 81% (Supplementary File 2a). Background, objectives, and randomization method were adequately reported across studies, while details of methods relating to PROs and full protocols were poorly reported (Supplementary File 2b). Two of the included studies were not assessed for quality of reporting as they were non-randomized pilot studies.

## Discussion

This review identified existing interventions designed to address GI symptoms and summarized the evidence for their effectiveness. Comprehensive rehabilitation including psychoeducation, diet, and exercise has the potential to improve self-reported bowel symptoms and health behaviours of survivors following CRC surgery. Pelvic floor muscle exercise programs alone may help reduce faecal incontinence faster than routine post-operative care. Dietary education may also have positive effects on faecal incontinence. There were other interventions developed based on evidence in other patient populations, such as probiotic and pharmacological supplements, which did not yield a significant improvement in bowel symptoms [20, 22].

Bowel symptoms are among the most bothersome and commonly reported consequences of primary treatment for CRC [7]. Inability to evacuate bowel in under 15 min and faecal incontinence remain highly prevalent more than two years post-treatment [7]. Furthermore, survivors report greater tendency to self-manage their symptoms, especially those that remain more than 1–2 years post-treatment [10]. CRC survivors have also reported feeling alone after diagnosis and primary treatment for CRC [23]. Although evidence is limited, findings from this review suggest that PFME may support earlier resolution of some GI symptoms. Comprehensive rehabilitation programs, particularly when administered early, may help survivors better navigate bowel symptoms at home, as they arise at different timepoints after treatment.

To our knowledge, there are ongoing studies evaluating interventions for GI symptoms: exercise interventions and a dietary education program. Exercise RCTs are evaluating the effectiveness of PFME programs on improving bowel function and severity of low anterior resection syndrome (LARS) symptoms such as faecal incontinence, frequency or urgency of stools, incomplete bowel movements, or tenesmus [24]. A large-scale RCT is currently underway for the telehealth dietary intervention assessed previously for feasibility [21], including bowel function and LARS as primary outcomes [25]. These ongoing studies will provide further intervention effectiveness evidence.

There are limitations to be noted in this review. Due to the small sample size and the number of studies identified, we were unable to make statistical comparisons between similar intervention types. The heterogeneity of PROMs used to assess outcomes also made it difficult for any meaningful comparisons between interventions targeting the same outcome. Furthermore, we only included studies that were published in English and excluded conference proceedings, so other relevant interventions may be available. There are services that exist within some local healthcare districts addressing bowel problems or healthcare professionals such as pelvic floor therapists and dieticians who specialize in CRC survivorship. However, our review may not have captured these due to a lack of published research evaluating these interventions.

## Conclusion

Despite high prevalence and burden of GI symptoms in CRC survivors, there is heterogeneity of types of interventions designed to address GI symptoms in CRC survivors, and limited evidence supporting the effectiveness of any type. Various interventions can be available but may not be effective for all patients, and consequently, some trial-and-error may be inevitable until further research is able to identify effective interventions for managing GI symptoms that occur after completing treatment for CRC.

## Supplementary information

ESM 1 (DOCX 175 kb)
