# Supplementary material for: Supportive care interventions for managing gastrointestinal symptoms following treatment for colorectal cancer: a systematic review
Source: J Cancer Surviv. 2023 Jun 6;18(5):1640–7. doi: 10.1007/s11764-023-01403-3 (PMC11424733; doi:10.1007/s11764-023-01403-3)

Supplementary file 1. Search strategy

| 1. (colorectal adj1 (cancer$ or neoplasm$ or tumo$r$ or carcinoma$)).ti,ab,mp. |
| --- |
| 2. (bowel adj1 (cancer$ or neoplasm$* or tumo$r$ or carcinoma$)).ti,ab,mp. |
| 3. (colon adj1 (cancer$ or neoplasm$ or tumo$r$ or carcinoma$)).ti,ab,mp. |
| 4. ((rectal or rectum or anal) adj1 (cancer$ or neoplasm$ or tumo$r$ or carcinoma$)).ti,ab,mp. |
| 5. (anal adj1 (cancer$ or neoplasm$ or tumo$r$ or carcinoma$)).ti,ab,mp. |
| 6. or/1-5 |
| 7. (patient reported outcome$ or PRO or PROM).ti,ab,kw. |
| 8. ((quality adj2 life) or QOL or HRQOL or HRQL).ti,ab. |
| 9. (health adj1 (status or related)).ti,ab. |
| 10. pain.ti,ab. |
| 11. sleep.ti,ab. |
| 12. (fatigue or tired*).ab,ti. |
| 13. (stress or distress).ti,ab. |
| 14. depression.ti,ab. |
| 15. (anxiety or worry).ti,ab. |
| 16. (fear adj5 recur*).ab,ti. |
| 17. physical function*.ti,ab. |
| 18. psychosocial.ti,ab. |
| 19. sexual*.ab,ti. |
| 20. (diarrhea or diarrhoea).ab. |
| 21. nausea.ab. |
| 22. vomit*.ab. |
| 23. constipat*.ab. |
| 24. f$ecal leakage.ab. |
| 25. f$ecal urgency.ab. |
| 26. urin$ leakage.ab. |
| 27. urin$ urgency.ab. |
| 28. (swell* or cramp*).ab. |
| 29. symptom*.ti,ab. |
| 30. gastrointestinal.ab,ti. |
| 31. gut.ab. |
| 32. appetite.ab. |
| 33. anorexia.ab. |
| 34. food sensitivit*.ti,ab. |
| 35. fertility.ti,ab. |
| 36. return to work.ti,ab. |
| 37. neuropathy.ti,ab. |
| 38. (skin and (irritation or breakdown)).ti,ab. |
| 39. incontinence.ti,ab. |
| 40. (gas or flatulence or flatus).ti,ab. |
| 41. (odour or smell).ti,ab. |
| 42. physical activit*.ti,ab. |
| 43. daily activit*.ti,ab. |
| 44. social activit*.ti,ab. |
| 45. social function*.ti,ab. |
| 46. role function*.ti,ab. |
| 47. isolation.ti,ab. |
| 48. body image.ti,ab. |
| 49. QLQ-C30.ti,ab,tw. |
| 50. QLQ-CR29.ti,ab,tw. |
| 51. FACT-G.ti,ab,tw. |
| 52. FACT-C.ti,ab,tw. |
| 53. FCSI.ti,ab,tw. |
| 54. or/7-53 |
| 55. random* controlled trial.mp,pt. |
| 56. (random$ or placebo$ or single blind$ or double blind$ or triple blind$).ti,ab. |
| 57. random* sampl$.ti,ab. |
| 58. experimental stud*.ti,ab. |
| 59. experimental trial*.ti,ab. |
| 60. random* effect$.ti,ab. |
| 61. clinical trial.ti,ab. |
| 62. non random*.ti,ab. |
| 62. evaluation stud*.ti,ab. |
| 64. evaluation trial*.ti,ab. |
| 65. non-random*.ti,ab. |
| 66. quasi experiment*.ti,ab. |
| 67. single arm.ti,ab,kw. |
| 68. single-arm.ti,ab,kw. |
| 69. clinical stud*.ti,ab. |
| 70. comparative stud*.ti,ab. |
| 71. comparative trial*.ti,ab. |
| 72. or/55-71 |
| 73. intervention$.ti,ab,kw. |
| 74. resource$.ti,ab,kw. |
| 75. service$.ti,ab,kw. |
| 76. (online or e*health).ti,ab,kw. |
| 77. telehealth.ti,ab,kw. |
| 78. Complimentary.ti,ab,kw. |
| 79. or/73-78 |
| 80. (p$ediatric* or child* or infant* or youth* or adolescen*).ti,ab. |
| 81. animal*.mp,ti,ab. |
| 82. (mouse or rat or mice).ab. |
| 83. (in vivo or in vitro).mp. |
| 84. ((semi-structur* or semistructur* or unstructur* or informal or in-depth or indepth or face-to-face or structur* or guide) adj3 (interview* or discussion* or questionnaire*)).ti,ab. |
| 85. interviews as topic/ |
| 86. (focus group* or qualitative or ethnograph* or fieldwork or "field work" or "key informant").ti,ab. |
| 87. focus groups/ or narration/ or qualitative research/ |
| 88. (retraction of publication or retracted publication).pt. |
| 89. retrospective.ti,ab. |
| 90. Epidemiolog*.ti,ab. |
| 91. cross-section*.ti,ab. |
| 92. Case Control Study/ or Control Group/ |
| 93. (case$ and control$).mp,tw. |
| 94. (case$ and series).tw. |
| 95. Matched-Pair Analysis/ |
| 96. (animal* not human*).sh. |
| 97. ((comment or editorial or meta-analysis or practice-guideline or review or letter) not random* control* trial).pt. |
| 98. (meta-analys$ or meta analys$ or metaanalys$).tw,sh,pt. |
| 99. ((systematic$ adj5 review$) or (systematic$ adj5 overview$) or (quantitativ$ adj5 review$) or (quantitativ$ adj5 overview$) or (quantitativ$ adj5 synthesis$) or (methodologic$ adj5 review$) or (methodologic$ adj5 overview$)).tw,sh. |
| 100. (integrative research review$ or research integration).tw. |
| 101. historical article.pt. |
| 102. review.pt. |
| 103. letter.pt. |
| 104. comment.pt. |
| 105. editorial.pt. |
| 106. ((clinical adj3 pathway) or (clinical adj3 pathways) or (practice adj3 parameter) or (practice adj3 parameters) or (algorithm* or care pathway) or care pathways).ti,ab,kw. |
| 107. Clinical protocols/ or Consensus/ |
| 108. Consensus Development Conference.pt. |
| 109. Consensus Development Conference*, NIH.pt. |
| 110. Consensus Development Conferences as Topic/ |
| 111. Consensus Development Conferences, NIH as Topic/ |
| 112. critical pathway/ |
| 113. guidance.ti,ab. |
| 114. guideline*.ti. |
| 115. guideline/ |
| 116. or/80-115 |
| 117. 6 and 54 |
| 118. 117 and 72 |
| 119. 118 and 79 |
| 120. 119 not 116 |

Supplementary file 2a. Quality assessment (%) by study

Supplementary file 2b. Quality assessment (%) by CONSORT-PRO items


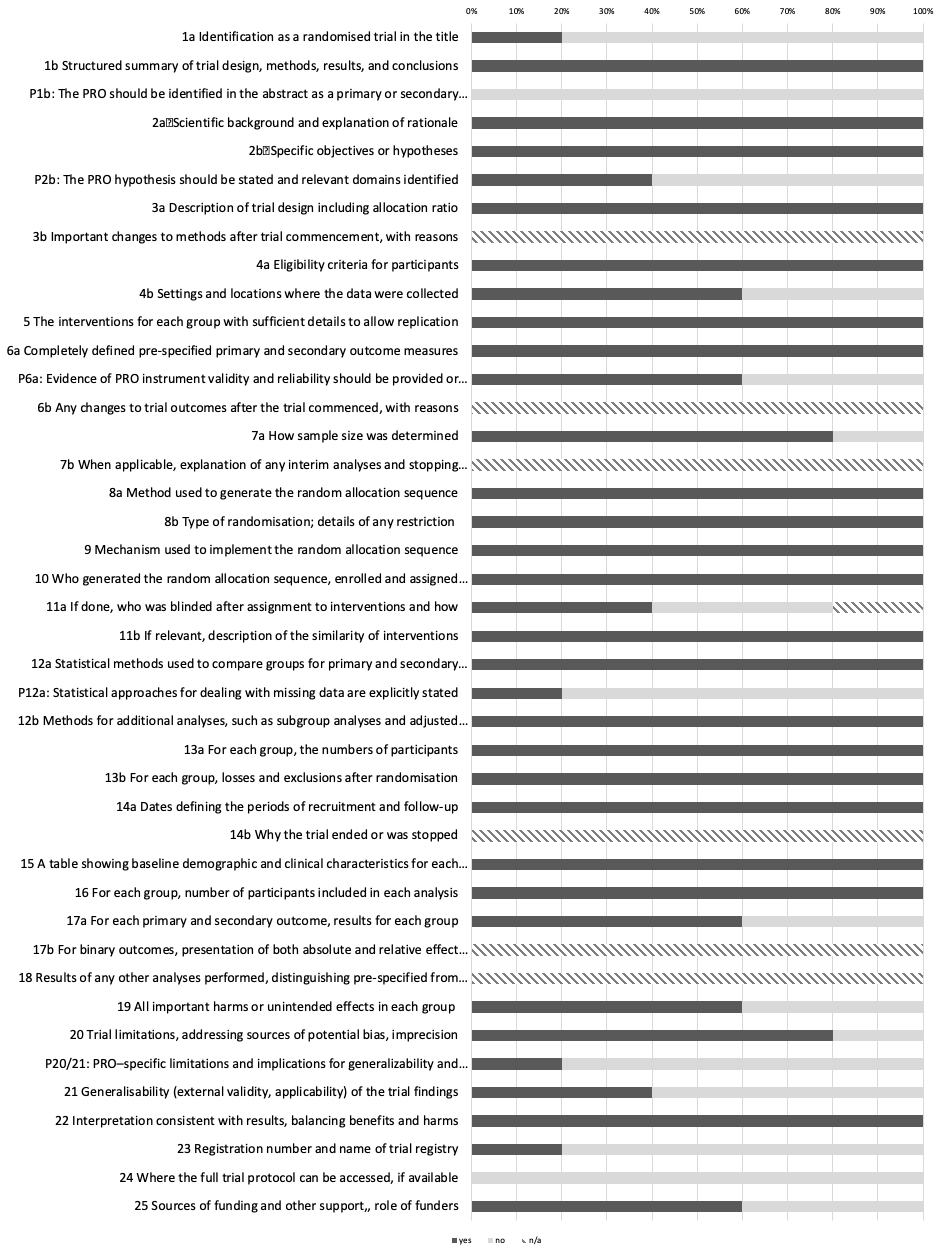

Supplement: Supplementary file 1 — (DOCX 175 kb) [file 11764_2023_1403_MOESM1_ESM.docx]
